# Pattern-Recognition Receptor Signaling Regulator mRNA Expression in Humans and Mice, and in Transient Inflammation or Progressive Fibrosis

**DOI:** 10.3390/ijms140918124

**Published:** 2013-09-04

**Authors:** Roman Günthner, Vankayala Ramaiah Santhosh Kumar, Georg Lorenz, Hans-Joachim Anders, Maciej Lech

**Affiliations:** Medical Clinic and Policlinic IV, Nephrology Center, University of Munich, 80336 Munich, Germany; E-Mails: santosh.kumar@med.uni-muenchen.de (V.R.S.K.); georg.lorenz@med.uni-muenchen.de (G.L.); hans-joachim.anders@med.uni-muenchen.de (H.-J.A.); maciej.lech@med.uni-muenchen.de (M.L.)

**Keywords:** inflammation, Toll-like receptors, infection, fibrogenesis, atrophy, pattern recognition receptors, chronic disease, innate immunity

## Abstract

The cell type-, organ-, and species-specific expression of the pattern-recognition receptors (PRRs) are well described but little is known about the respective expression profiles of their negative regulators. We therefore determined the mRNA expression levels of *A20*, *CYLD*, *DUBA*, *ST2*, *CD180*, *SIGIRR*, *TANK*, *SOCS1*, *SOCS3*, *SHIP*, *IRAK-M*, *DOK1*, *DOK2*, *SHP1*, *SHP2*, *TOLLIP*, *IRF4*, *SIKE*, *NLRX1*, *ERBIN*, *CENTB1*, and *Clec4a2* in human and mouse solid organs. Humans and mice displayed significant differences between their respective mRNA expression patterns of these factors. Additionally, we characterized their expression profiles in mononuclear blood cells upon bacterial endotoxin, which showed a consistent induction of *A20*, *SOCS3*, *IRAK-M*, and *Clec4a2* in human and murine cells. Furthermore, we studied the expression pattern in transient kidney ischemia-reperfusion injury versus post-ischemic atrophy and fibrosis in mice. *A20*, *CD180*, *ST2*, *SOCS1*, *SOCS3*, *SHIP*, *IRAK-M*, *DOK1*, *DOK2*, *IRF4*, *CENTB1*, and *Clec4a2* were all induced, albeit at different times of injury and repair. Progressive fibrosis was associated with a persistent induction of these factors. Thus, the organ- and species-specific expression patterns need to be considered in the design and interpretation of studies related to PRR-mediated innate immunity, which seems to be involved in tissue injury, tissue regeneration and in progressive tissue scarring.

## 1. Introduction

Pattern-recognition receptors (PRRs) are germ-line encoded receptors of the innate immune system that translate the recognition of pathogen-associated molecular patterns (PAMPs) and tissue damage-associated molecular patterns (DAMPs) into an immediate and antigen-unspecific inflammatory response [1]. Some of the generally known PRRs are Toll-like receptors, RIG-I-like helicases and NOD-like receptors. TLR expression is induced in injured tissues, e.g., during post-ischemic sterile inflammation of the kidney, which largely refers to the recruitment of different leukocyte subsets but also to cytokine-related induction of TLR expression in parenchymal tissue cells [2]. TLR activation induces the recruitment of intracellular signaling adaptors that interact with the TLR’s intracellular TIR domain by TIR-TIR domain interactions, conceptually similar to the IL-1Rs [1]. Downstream of these adaptors, a set of kinases and co-factors integrate the signals for the activation of transcription factors, such as NF-κB and interferon-responsive factors that induce the expression of numerous pro-inflammatory mediators. A tight regulation of PRR signaling is necessary to avoid overshooting inflammation [3,4], which can be harmful by itself, e.g., in early sepsis (cytokine storm), in pneumonia or meningitis [5]. Sometimes inflammation is generally inappropriate as host defense is not at all required in sterile injuries, e.g., in ischemia-reperfusion- or toxin-related tissue injury [6]. Hence, immunoregulatory elements exist at all levels of the outside-in PRR signaling pathways [7,8].

Regulators of the signaling cascade can appear as enzymes, orphan receptors and adaptor proteins. A20 is a deubiquitinase that inhibits TRAF6 downstream of TLR2, -3, -4, and -9 in macrophages and thereby protects mice from endotoxic shock [9,10]. It also interferes with RIG-I/MDA5 and NOD2 signaling [9,10]. The ubiquitin-editing enzyme CYLD inhibits TRAF6 and -7 and TLR2 [11]. DUBA is a deubiquitinase that targets TRAF3 and inhibits IFN-β secretion downstream of RIG-I/MDA-5 and TLRs [12]. ST2 is a member of the TIR domain-containing family that inhibits TLR2 as well as TLR4 signaling and is also crucial for the development of endotoxin tolerance [13,14]. CD180 is a TLR4 homologue that interferes with LPS binding to the TLR4/MD-2 complex in peritoneal macrophages and DCs [15]. SIGIRR (also TIR8), an orphan receptor that interacts with TRAF6, IL-1-R1, and IRAK, inhibits signaling downstream of the IL-1R and TLR2, -3, -4, -7 and -9 [16,17]. TANK suppresses TLR signaling by inhibiting the ubiquitination of TRAF6 [18]. Suppressor of cytokine signaling (SOCS)-1 interferes with JAK signaling and is essential for the control of LPS-induced inflammation [19]. In contrast, SOCS3 interferes with JAK/STAT signaling, IL-1 signaling and prevents macrophages from M1 polarization, which leads to aggravated tissue damage in *Socs3*-deficient mice [20,21]. “SH2-containing inositol-5-phosphatase” (SHIP or SHIP1) is important for endotoxin tolerance and negatively regulates TLR4 and TLR3 signaling [22,23]. IL-1R-associated kinase (IRAK)-M, also named IRAK-3, suppresses signaling of several TLR subtypes, especially TLR9. It also contributes to the phenomenon of endotoxin tolerance [24]. Downstream of tyrosine kinases (DOK1 and DOK2) are adaptor proteins that prevent specifically prevent ERK activation through TLR4 stimulation [25]. SH2-containing protein tyrosine phosphatase (SHP)-1 is a tyrosine phosphatase targeting IRAK-1 that suppresses NF-κB activation in splenocytes, dendritic cells (DCs) and macrophages [26]. The related SHP-2 directly interacts with TBK1 and thus negatively regulates the TRIF-mediated IFN-β production downstream of TLR3 and TLR4 signaling in macrophages [27]. Toll-interacting protein (TOLLIP) that interacts with IRAK is described as a negative regulator of TLR2, -4 and IL-1-signaling in human monocytes [28]. IRF-4 is a transcription factor that interacts with MyD88 and suppresses TLR-dependent secretion of pro-inflammatory cytokines in macrophages [29]. Suppressor of IKKɛ (SIKE) specifically interferes with IFN-β production following TLR3 and RIG-I stimulation [30]. NLRX1, a NOD-like receptor family member interacting with TRAF 6 and IKK was recently described as a negative regulator of RIG-I/MAVS and TLR4 signaling [31,32]. Erbin is a specific negative regulator of the NOD2-pathway via direct interaction with NOD2 [33]. CENTB1 specifically inhibits NOD1 and NOD2 signaling [34]. CLEC4A2 (also CLECSF6 or DCIR), a C-type lectin receptor bears an inhibitory ITIM motif and plays a crucial role in regulation of DCs, but can also suppress cytokine secretion following TLR8 stimulation [35,36].

Species-specific differences in inflammation-related gene expression clearly exist [37]. As long as animal models remain the tool of choice in many areas of immunology research, a deeper knowledge about such differences is important to guide data interpretation and making suitable predictions about human immunity. As expression patterns of the PRRs differ among species, we hypothesized the same for their signaling regulators. Hence, we determined their mRNA expression profiles in human and murine organs as well as during tissue regeneration upon transient injury versus progressive tissue atrophy and fibrosis.

## 2. Results and Discussion

### 2.1. PRR Signaling Regulator mRNA Expression in Adult Human Tissues

**Figure 1:**
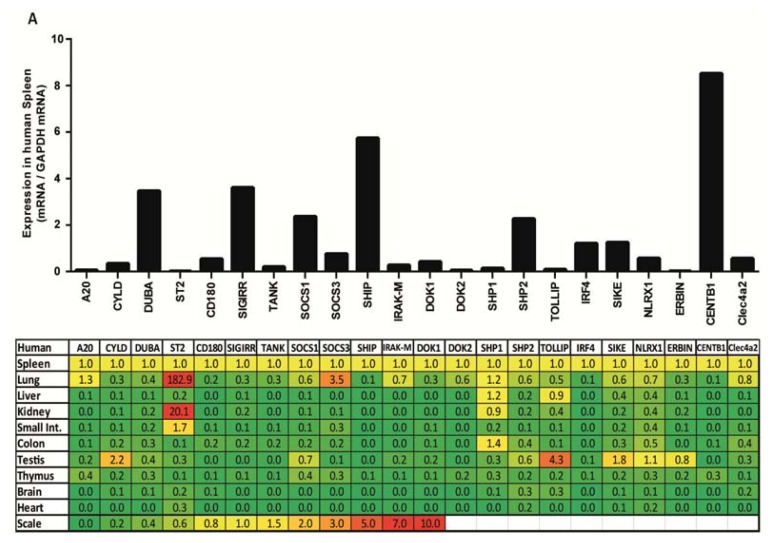

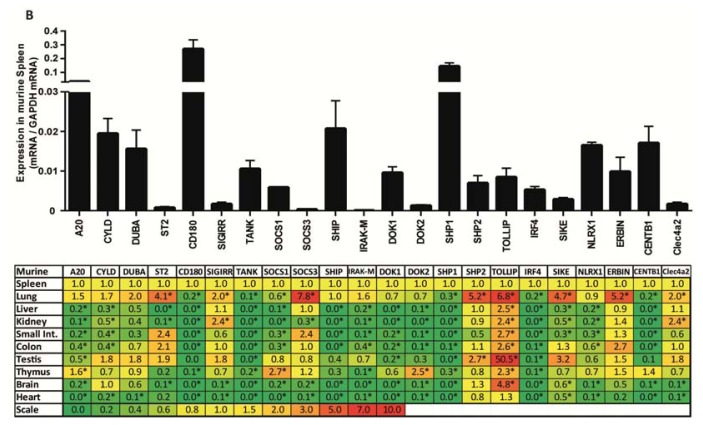
Pattern-recognition receptor (PRR) negative regulators mRNA expression in adult human and mouse tissues. (**A**) Basal mRNA expression of negative regulators in human tissues. Quantitative real-time PCR analysis of pre-normalized cDNA derived from poly-(A)-selected DNase-treated RNA isolated from 10 several tissues was performed as described in experimental section. mRNA expression levels were calculated using human *Glyceraldehyd-3-Phosphat-Dehydrogenase* (*GAPDH)* as a housekeeping gene. Spleen was chosen as a reference organ. Spleen mRNA expression levels are shown in the upper graph. Expression of the genes in other human organs is indicated in the table as x-fold induction (or suppression) compared to expression in spleen. Yellow shades illustrate similar, red colors increased and green colors decreased mRNA levels; (**B**) Basal mRNA expression of negative regulators in murine tissues. Quantitative real-time PCR analysis of cDNA derived from RNA isolated from 10 several murine (C57BL/6) tissues was performed as described in experimental section. Detected mRNA expression levels were calculated using murine *Glyceraldehyd-3-Phosphat-Dehydrogenase* (*GAPDH)* as a housekeeping gene; spleen mRNA expression levels are illustrated in the upper graph; error bars represent SEM. Expression of the genes in other murine organs is indicated in the table as x-fold induction (or suppression) compared to expression in spleen. Yellow shades illustrate similar, red colors increased and green colors decreased mRNA levels.

We used real time quantitative real time polymerase chain reaction (qRT-PCR) to quantify the mRNA expression levels of the following PRR signaling regulators in human solid organs: *A20*, *CYLD*, *DUBA*, *ST2*, *CD180*, *SIGIRR*, *TANK*, *SOCS1*, *SOCS3*, *SHIP*, *IRAK-M*, *DOK1*, *DOK2*, *SHP1*, *SHP2*, *TOLLIP*, *IRF4*, *SIKE*, *NLRX1*, *ERBIN*, *CENTB1* and *Clec4a2*. All of these molecules were constitutively expressed in human spleen but the mRNA expression levels of *A20*, *ST2*, *DOK2*, and *ERBIN* were low (Figure 1A). Generally, the expression of most aforementioned factors was lower in the nine solid organs tested as compared to spleen. However, *ST2* expression was 182-fold higher in lung and 20-fold higher in kidney. In addition, *SOCS3* expression was 3.5-fold higher in lung and *TOLLIP* expression was 4.3-fold higher in testis. Thus, the mRNA expression levels of most PRR signaling regulators are low in healthy solid organs compared to spleen, except for *ST2*, which is low in spleen but high in lung and kidney.

### 2.2. PRR Signaling Regulator mRNA Expression in Adult Murine Tissues

**Figure 2:**
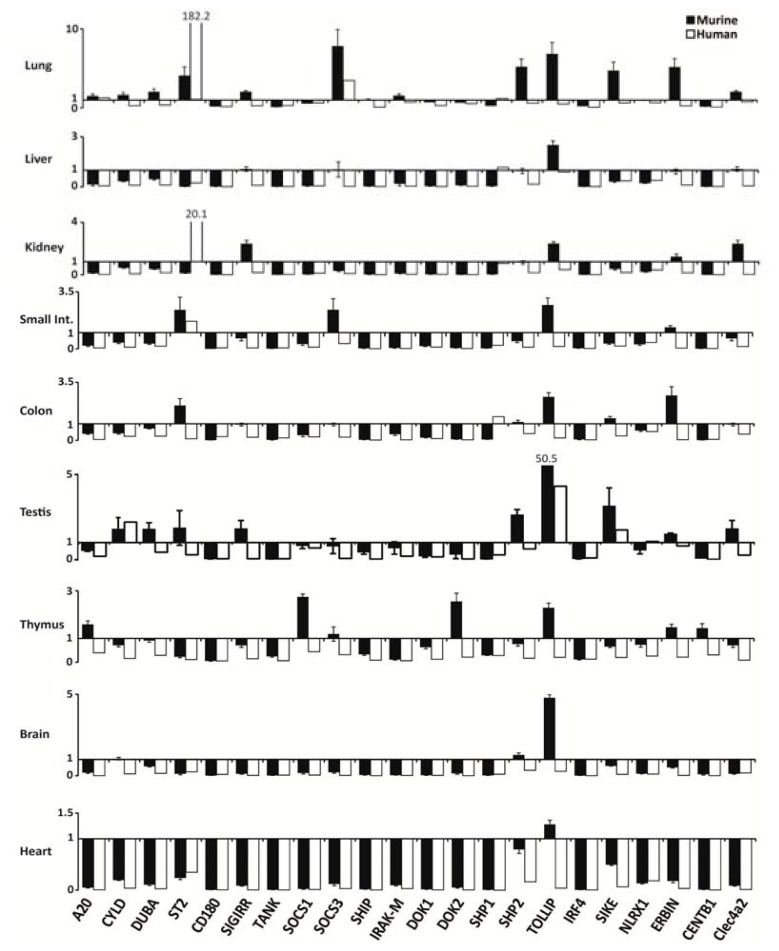
Interspecies comparison of relative expression of PRR negative regulators in different organs compared to spleen. The respective relative murine (black bars) and human (open bars) PRR negative regulators mRNA levels from Figure 1A and 1B are illustrated to directly compare expression between mice and humans. The *y*-axis marks the fold-change in each direction, whereas *x*-axis marks the different genes used in the analysis. Note that the scale of the *y*-axis is different for each organ. Data represent means ± SEM.

Next, we determined the mRNA expression levels of the same PRR signaling regulators in the same organs from 12 week old C57BL/6 mice. All molecules were constitutively expressed in mouse spleen but the mRNA levels of *SOCS3* and *IRAK-M* were low (Figure 1B). Similar to human solid organs the PRR regulator mRNA levels were much lower than in mouse spleen except for the following: Lung expressed higher levels of *ST2*, *SOCS3*, *SHP2*, *TOLLIP*, *SIKE*, and *ERBIN. TOLLIP* levels were also higher in all other organs, especially in testis. Figure 2 compares the organ-specific PRR signaling regulator mRNA expression levels in humans and mice where white and black (murine) bars indicate the x-fold induction versus respective spleen mRNA levels. The signatures were mostly concordant, e.g., in liver, brain, and heart. However, *TOLLIP* mRNA expression was discordant in most organs with higher levels in mouse organs, while most human organs displayed lower *TOLLIP* mRNA levels compared to spleen. In addition, *ST2* expression was discordant in kidney, colon, and testis and showed much higher relative levels in human lung. Thus, the relative human and mouse mRNA expression levels of PRR signaling regulators in solid organs are not always consistent, especially those of *TOLLIP* and *ST2.*

### 2.3. PRR Signaling Regulator mRNA Expression upon Bacterial Endotoxin Exposure

**Figure 3:**
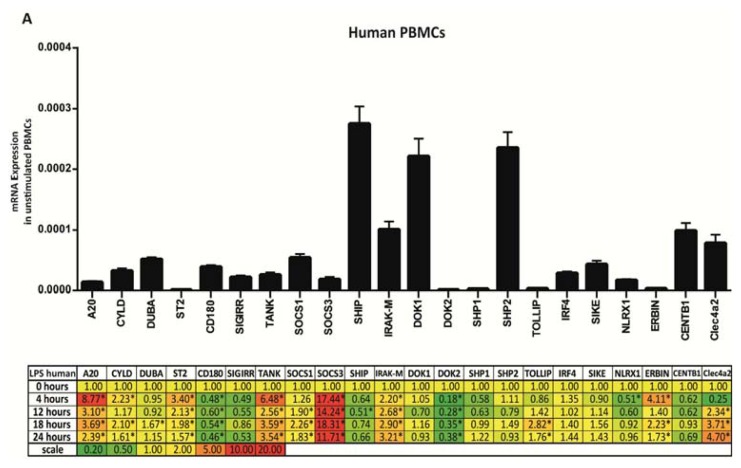

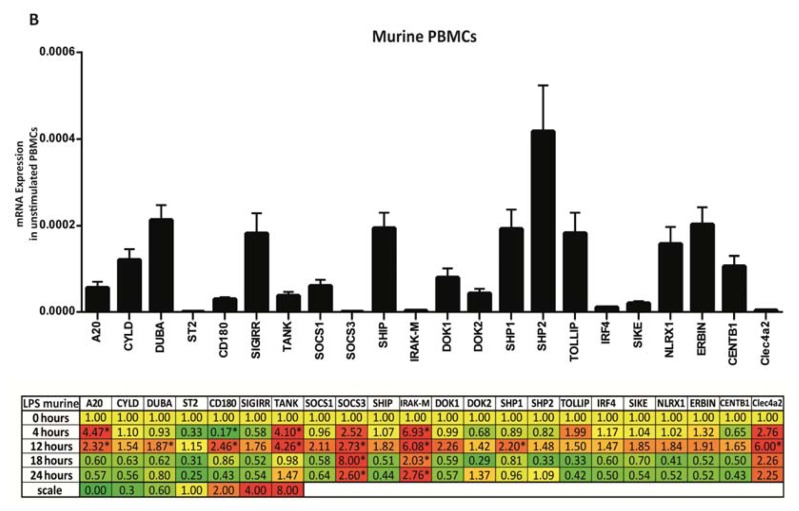
Expression levels of peripheral blood mononuclear cells (PBMCs) stimulated with LPS. (**A**) mRNA expression in human PBMCs; (**B**) mRNA expression in murine PBMCs. PBMCs were isolated from humans/mice and stimulated with 500 ng/mL LPS for 4, 12, 18 and 24 h as described in experimental section. Histograms show the basal expression in PBS treated controls. *18S* served as a housekeeping gene in both, humans and mice to remain comparability. Expression of the genes at chosen time points is indicated in the table as x-fold induction (or suppression) compared to controls. Yellow shades illustrate similar, red colors increased and green colors decreased mRNA levels.

The phenomenon of endotoxin tolerance is largely based on the induction of negative regulators of TLR4 signaling [38]. Therefore, we studied the induction of all 22 PRR signaling regulators in human and mouse peripheral blood mononuclear cells (PBMCs) after 4, 12, 18, and 24 h of exposure to bacterial endotoxin/LPS. In human PBMCs *A20*, *CYLD*, *ST2*, *TANK*, *SOCS3*, *IRAK-M*, *ERBIN*, and *Clec4a2* were induced more than 2-fold as early as 4 h upon stimulation, while *SOCS1* and *Clec4a2* were significantly induced only at 12 h (Figure 3A). Only *DUBA* and *TOLLIP* came in at 18 h, but their induction was mild. *A20*, *TANK*, *SOCS3* and *IRAK-M* were the only genes to be significantly induced at all time points. The same mRNA expression pattern was found in human PBMCs stimulated with only 10 ng/mL LPS (Figure S1). In murine PBMCs *A20*, *TANK*, *SOCS3*, *IRAK-M*, and *Clec4a2* were induced more than 2-fold as early as 4 h upon stimulation, while *DUBA*, *CD180*, and *SHP1* were significantly induced only at 12 h (Figure 3B). No additional genes came in at later time points. *SOCS3* and *IRAK-M* were the only genes to be significantly induced at all time points studied, while all other induced factors where only transiently induced and went back to baseline at 18 and 24 h (Figure 3B). Thus, bacterial endotoxin consistently induces *A20*, *SOCS3*, *IRAK-M*, and *Clec4a2* in human and mouse PBMCs, but the onset and duration of PRR signaling regulator expression is somewhat different in human and mouse PBMCs.

### 2.4. PRR Signaling Regulator mRNA Expression in Transient Ischemia-Reperfusion Injury

**Figure 4:**
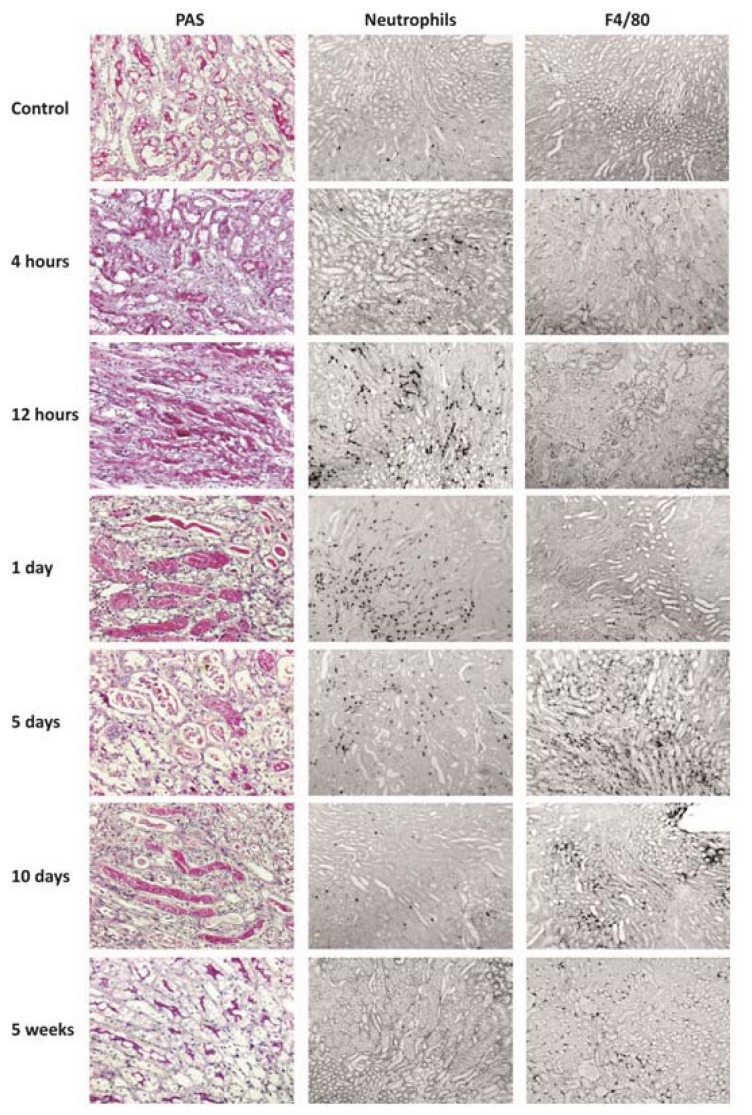
Tissue histology upon kidney ischemia-reperfusion. Renal ischemia-reperfusion injury was induced as described in experimental section. Representative images of renal sections stained with PAS, Neutrophil staining for neutrophils or F4/80 for macrophages are shown at 6 time points. Original magnification: 200× for PAS and 100× for neutrophils and F4/80.

**Figure 5:**
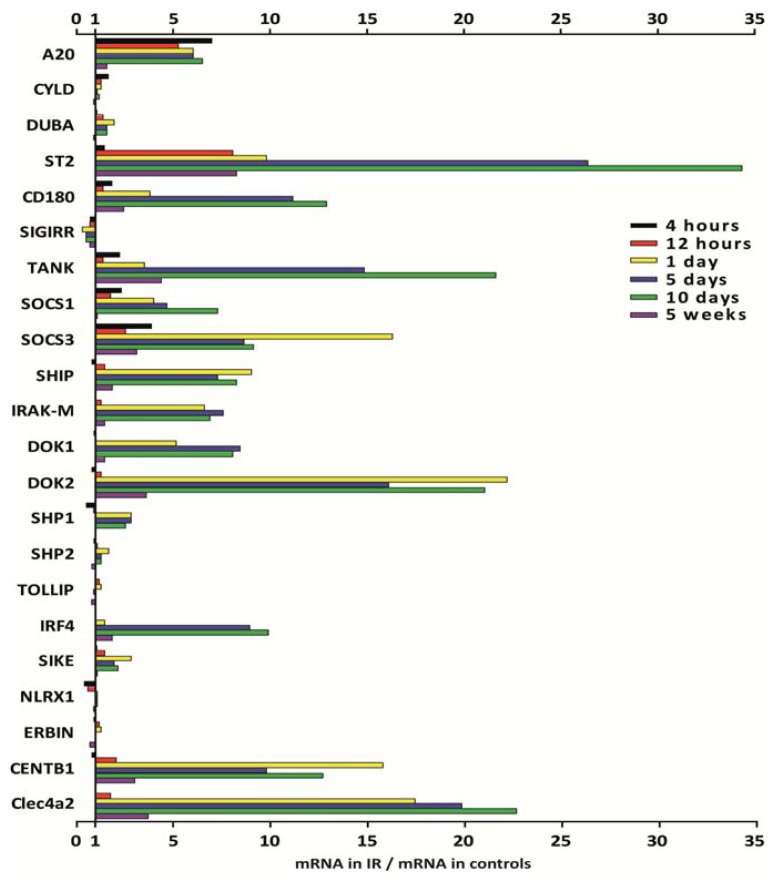
PRR negative regulators mRNA expression in the kidney undergoing ischemia-reperfusion injury. Renal ischemia-reperfusion injury was induced by clamping the renal artery for 45 min and organs were harvested after 6 different timepoints as described in experimental section. RNA was isolated from injured kidneys or contralateral controls and transcribed into cDNA. qRT-PCR was performed and mRNA expression was determined using *18S* as a housekeeping gene. Expression levels are illustrated in the bar graph as x-fold induction compared to contralateral kidneys, which served as controls.

Tissue injuries often involve sterile inflammation triggered by DAMPs that have the potential to activate PRR signaling just as PAMPs. Therefore, we intended to study transient versus progressive sterile tissue inflammation. We selected ischemia-reperfusion injury upon renal pedicle clamping because this model is associated with a transient TLR2/4/MyD88-mediated sterile inflammation at day 1 in association with neutrophil infiltrates (Figure 4) [39,40]. At this time point the mRNA expression levels of *A20*, *ST2*, *SOCS3*, *SHIP*, *IRAK-M*, *DOK1*, *DOK2*, *CENTB1*, and *Clec4a2* were induced more than 5-fold above baseline (Figure 5). It is of note that only *A20* was induced as early as 4 h and *ST2* from 12 h upon renal pedicle clamping. The subsequent resolution of inflammation goes along with epithelial regeneration, which is associated with disappearance of neutrophils and a transient influx of alternatively-activated macrophages that support the healing process (Figure 4) [41–43]. Most of the aforementioned factors that were induced at day 1 remained induced also at day 5 and 10, especially *ST2* and *Clec4a2* mRNA levels increased with time. *CD180*, *TANK*, *SOCS1*, and *IRF4* were induced only from day 5 (Figure 5). Five weeks after renal pedicle clamping the kidney had completely regenerated, which was associated with a mRNA level decline of most of the once induced factors, except for *ST2* that remained elevated 8.5-fold above baseline. *CYLD*, *DUBA*, *SHP1*, *SHP2*, *TOLLIP*, *SIKE*, *NLRX1*, and *ERBIN* were not at all or hardly regulated throughout this transient disease process. Together, transient sterile inflammation induces *A20*, *CD180*, *ST2*, *SOCS1*, *SOCS3*, *SHIP*, *IRAK-M*, *DOK1*, *DOK2*, *IRF4*, *CENTB1*, and *Clec4a2* albeit at different phases of the injury and repair process.

### 2.5. PRR Signaling Regulator mRNA Expression in Progressive Tissue Fibrosis

**Figure 6:**
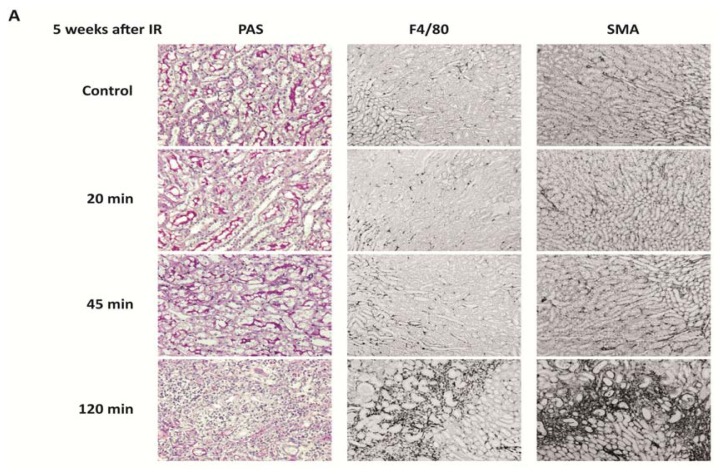

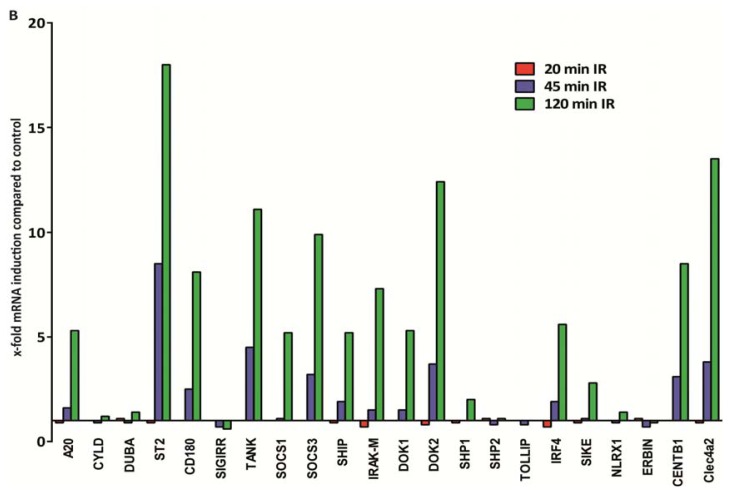
Histology and PRR negative regulators mRNA expression in kidneys upon different ischemia times. (**A**) Renal ischemia-reperfusion injury was induced by clamping the renal artery for 20, 45 and 120 min and organs were harvested after five weeks as described in experimental section. Representative images of renal sections were stained with PAS, F4/80 as a macrophage marker and SMA as a fibrosis marker. Original magnification: ×200 for PAS and 100× for F4/80 and SMA; (**B**) RNA was isolated from injured kidneys or contralateral controls, transcribed into cDNA and qRT-PCR was performed. *18S* served as a housekeeping gene. Induction of negative regulators five weeks after IRI is shown as x-fold induction compared to contralateral kidneys.

The capacity for postischemic tissue regeneration depends on the extent of the initial injury. Extensive damage may also include loss of those progenitor stem and progenitor cells that account for the regenerative process [44]. In our experimental system, this can be mimicked by different durations of renal pedicle clamping. For example, ischemia time of 20 min does not cause significant kidney injury, while 45 min of ischemia causes transient injury and inflammation with full recovery within five weeks (Figure 6A). Accordingly, 20 min of ischemia was not associated with a significant regulation of any of the PRR signaling modulators five weeks later (Figure 6B). As described before, 45 min of ischemia was associated only with a persistent induction of *ST2*, even though many genes had been induced during the transient disease process. In contrast, 120 min of ischemia causes extensive injury, persistent tubular atrophy, and progressive renal fibrosis (Figure 6A), which was associated with a persistent induction of *A20*, *ST2*, *CD180*, *TANK*, *SOCS1*, *SOCS3*, *SHIP*, *IRAK-M*, *DOK1*, *DOK2*, *IRF4*, *CENTB1*, and *Clec4a2* (Figures 6B). This pattern was similar to that of the repair phase upon 45 min of ischemia (Figure 4). Thus, progressive tissue fibrosis following up on severe ischemia-reperfusion injury is associated with a persistent induction of those PRR signaling regulators that are induced during the recovery phase from transient injury.

### 2.6. Negative Regulators of PRRs in Inflammation and Tissue Homeostasis

PRR signaling triggers innate immunity in infectious and non-infectious forms of tissue inflammation. Counterbalancing factors are important to avoid a potentially harmful “cytokine storm” and to limit the duration of PRR signaling, a prerequisite also for the resolution of inflammation upon transient triggers. Our data demonstrate an organ- and species-specific expression pattern of the PRR signaling regulators. Only some of them are rapidly and persistently induced upon exposure to bacterial toxin in PBMCs. These and other regulators are induced in post-ischemic tissues, which may relate to the sequential recruitment of different leukocyte subsets. Progressive tissue remodeling and scaring upon severe acute injury are associated with a persistent induction of those PRR signaling regulators that are expressed during the repair phase of acute injuries.

We found several differences in the relative mRNA expression profiles of the PRR signaling regulators in mice and humans, similar to what has been described for the TLRs [45], the NLRs, RLHs, and inflammasomes [46] as well as the C-type lectin receptors [47]. It remains a limitation of our study that the human cDNA samples were obtained from a small number of individuals, which might imply some selection bias. However, species-specific expression patterns need to be considered in the interpretation of either data and human studies need to verify the functional roles of single signaling regulators suggested by rodent studies.

Endotoxin tolerance is a well-known *in vitro* phenomenon describing that endotoxin-activated macrophages remain temporarily refractory to a second endotoxin stimulus [38]. This phenomenon is (partially) based on the induction of a number of signaling regulators mostly of TLRs that inhibit the signaling pathway at all levels [48]. We found a rather consistent induction of *A20*, *SOCS3*, *IRAK-M*, and *Clec4a2* in human and mouse PBMCs upon bacterial endotoxin exposure, which is consistent with previous studies that described some of these molecules to be involved in endotoxin tolerance [49–51]. The phenomenon of endotoxin tolerance has widespread clinical implications. It does not only account for systemic immunosuppression of advanced sepsis, a condition that largely accounts for ICU mortality in that phase [52], it also limits the extent of immunopathology and enhances the resolution of inflammation upon sterile injuries [3,53]. Therefore, we studied the regulation of PRR signaling inhibitors in transient versus progressive sterile tissue inflammation induced by ischemia-reperfusion injury.

Ischemia-reperfusion injury is characterized by a serial influx of neutrophils and pro-inflammatory macrophages that contribute to the inflammatory injury phase that lasts until day 2 upon renal pedicle clamping [41]. This phase is followed by a macrophage phenotype switch toward anti-inflammatory macrophages that rather contribute to the recovery of the injured tissue [41–43,54,55]. *A20* and *SOCS3* were induced as early as 4 h after clamping and both regulators remained induced until full recovery had occurred. Due to the serial appearance of different leukocytes in this model, it seems unlikely that this induction relates to a particular immune cell type [41]. *A20* and *SOCS3* are both expressed also by non-immune parenchymal cells and their induction may implicate the parenchyma’s attempt to minimize immunopathology [56,57]. It is noteworthy that *SIGIRR* was persistently downregulated throughout the injury and recovery phase, a finding that we had previously also demonstrated at the protein level [58]. *SIGIRR* is expressed by renal dendritic cells and tubular epithelial cells, although its inhibitory effect on TLR signaling only operates in the dendritic cells [59]. Nevertheless, *SIGIRR* was shown to limit post-ischemic renal inflammation and immunopathology in this and other models [58–62]. Similarly, *IRF4*, a known negative regulator of TLR signaling [29], was not significantly induced before five days after renal pedicle clamping at mRNA level and at protein level as shown previously by us [63]. However, *IRF4* is still functionally important to limit post-ischemic kidney injury as early as at 24 h [63]. These two examples demonstrate that the mRNA profiles predict also the respective protein expression and functional inhibition of TLR signaling in post-ischemic tissues. Obviously, the mRNA expression profiles do not always predict functional importance of this factor for the disease process. Nevertheless, it is interesting that factors like *ST2*, *TANK*, *DOK2*, *CENTB1*, and *Clec4a2* were consistently induced upon renal ischemia-reperfusion injury, at least *ST2* was shown to limit intestinal ischemia-reperfusion injury [64], hence, the functional contribution of the other induced genes should be tested.

We studied progressive tissue fibrosis using the same model of ischemia reperfusion injury by applying a longer ischemia time, which turned kidney recovery into kidney atrophy and scarring. There is an ongoing debate whether fibrogenesis is an independent pathomechanism of end stage (kidney) disease or whether fibrosis is rather a secondary healing response to stabilize the remaining parenchyma, whenever parenchymal healing is insufficient [65,66]. In fact, preventing fibrogenesis may not affect parenchymal loss and organ failure [67], and gene expression profiles taken from scarring allografts remain dominated by parenchymal injury markers [68]. Our results from the present study document a largely consistent expression profile of the PRR signaling inhibitors during progressive scarring and the (non-fibrotic) recovery phase of transient epithelial injury. This implies that the need for controlling inflammatory pathways is similar in epithelial and in mesenchymal repair. This is in line with the observation that anti-inflammatory immune cells dominate in the healing phase of transient injuries and in progressive fibrosis [54]. It is of note that the phenotype of the anti-inflammatory macrophages depends on *IRF4* [69], which we found to be induced in the recovery phase of acute kidney injury but also during progressive scaring in the post-ischemic kidney.

Together, we identified significant differences in the mRNA expression of PRR signaling regulators in human and mouse solid organs and in their regulation in transient inflammation and progressive tissue fibrosis. These findings can help to generate novel hypotheses on the role of single PRR signaling regulators in specific diseases. Furthermore, the species-specific expression of single PRR co-factors need to be considered in the interpretation of either data and human studies need to verify the functional roles of single factors suggested by studies performed in rodents.

## 3. Experimental Section

### 3.1. Human Solid Organ cDNA Preparation

Human solid organ prenormalized cDNA derived from poly-(A)-selected DNase-treated RNAs, which were purified from tissues of healthy male and female humans of caucasian descent were obtained from Clontech, Mountain View, CA. For RNA isolation, either whole organs or tissue samples representing the entire organ were used. To reduce the risk of selection bias, the purchased cDNA preparations are pools of cDNA from many human individuals for most of the organs as it is stated in the manufacturer’s certificate of analysis. (Table S1) An equal amount of cDNA from each preparation was used as a template in PCR. A 18S ribosomal unit was not detectable as it was not isolated in the poly-(A)-purified RNAs. Hence, we used *GAPDH/G3PDH* as the housekeeping gene for the analysis of human solid organ cDNA. The PCR product band was determined by video imaging and computer analysis, and band intensity was determined. If necessary, the concentration of individual cDNA preparations was than adjusted so that the average band intensity for the reference genes used to normalize the panel varied no more than 20%. As only a single pool was available for each organ, no studies on biological replicates allowing statistics could be performed. According to Clontech all human samples were purchased and imported in accordance with all local laws and regulations. Donors were tested to be negative for HIV, hepatitis B virus, and hepatitis C virus. Further exclusion criteria were as follows: manifest infections during the last 4 weeks, fever, symptomatic allergies, abnormal blood cell counts, increased liver enzymes, or medication of any kind except vitamins and oral contraceptives. The study was approved by the Ethics committee of Klinikum der Universität München and does not refer to any other experiments.

### 3.2. Mouse Solid Organ cDNA Preparation

Twelve week old adult C57BL/6 mice were purchased from Charles River, Sulzfeld, Germany and maintained under standard conditions and 12 h light/dark cycle. Animals were housed in polypropylene cages and allowed free access to food and water *ad libitum*. Mice were sacrificed by cervical dislocation, tissues were kept in RNAlater (Ambion, Carlsbad CA, USA) and high quality, DNA-free, RNA was isolated from same tissue mass (10 mg) with Pure Link RNA Mini Kit (Ambion, Carlsbad, CA, USA) according to manufacture instructions as described [46]. Samples were digested with DNAse solution and additional washing steps were performed to remove traces of DNAse. Concentrations of aqueous RNA samples were measured with NanoDrop 1000 Spectrophotometer (PEQLAB Biotechnologie, Erlangen, Germany). Only samples with absorbance 260/280 between 1.95 and 2.05 were considered as pure RNA, the integrity of the total RNA was determined by electrophoresis on 2% (*w*/*v*) agarose gels as described. 1 μg of good quality RNA of each individual sample was preceded to cDNA using thermo stable RNAse inhibitor during reverse transcription as described [47]. Reverse transcription was performed with a reaction mix containing Superscript II reverse transcriptase (Invitrogen, Grand Island, NY, USA), dNTPs, hexanucleotides, linear acrylamid, DTT and 5× Superscript buffers using standard protocol. cDNA synthesis reaction was performed for 90 min at 42 °C.

### 3.3. Animal Models of Transient and Progressive Tissue Inflammation

Groups of eight week old C57BL/6 mice (*n* = 5–10) underwent unilateral renal pedicle clamping for 45 min followed by reperfusion as a model of ischemia-reperfusion injury (IRI) as described [58]. Body temperature was maintained at 37 °C throughout the procedure by placing the mice on a heating pad. Mice were sacrificed 4 h, 12 h, 1 day, 5 days, 10 days and 5 weeks after the procedure. To investigate tissue fibrosis one of the renal arteries was clamped for 20, 45 or 120 min followed by reperfusion and sacrificing 5 weeks after surgery. Injured and contralateral kidneys were harvested for RNA isolation. Contralateral kidneys served as intraindividual controls. cDNA preparation was done with 1 μg per sample as described above. All experimental procedures were performed according to the German animal care and ethics legislation and had been approved by the local government authorities.

### 3.4. Quantitative Real-Time RT-PCR

*GAPDH* was chosen for analysis of the healthy human and murine tissues due to the lack of *18S* in human samples. Ribosomal protein *18S* was used as a reference gene for IRI mouse model and PBMC stimulations. Geometric mean (GM), arithmetic mean (AM) minimal value, maximal value, standard deviation (SD), variance and coefficient of variance (CV) of the housekeeping genes were calculated (Table S2). PRR signaling regulator mRNA expression in cDNAs of healthy organs, IRI kidneys and PBMC stimulations was quantified by real-time RT-PCR. Each PCR reaction (20 μL) (information) contained 10× Taq Polymerase Buffer, Taq Polymerase, dNTPs, BSA, PCR Optimizer, SYBR green solution, MgCl_2_, gene specific primers and 0.2 μL of synthesized cDNA. SYBR Green Dye detection system (SYBR Green I 96 protocol LC480 Roche running program, Roche, Penzberg, Germany) was used for amplification. Quantitative real-time PCR was performed on Light Cycler 480 (Roche, Mannheim, Germany). Each amplification step included initiation phase at 95 °C, annealing phase at 60 °C and amplification phase at 72 °C and was repeated 45 times. Gene-specific primers (300 nM, Metabion, Martinsried, Germany) were used as listed in Tables 1 and 2. Controls consisting of ddH_2_O were negative for target and housekeeping genes. Primers were designed to be cDNA specific and to target most CCDS approved transcripts. In silico specificity screen (BLAST) was performed. The lengths of amplicons were between 80 and 148 bp. The kinetics of the PCR amplification (efficiency) was calculated for every set of primers. The efficiency-corrected quantification was performed automatically by the Light Cycler 480 based on extern standard curves describing the PCR efficiencies of the target and the reference gene (ratio = *E*_target_*^ΔCP^*^target (control − sample)^/*E*_ref_*^ΔCP^*^ref (control − sample)^). To reduce the risk of false positive *Cp* the high confidence algorithm was used. All the samples that did not rise above the background fluorescence (crossing point *Cp* or quantification cycle *Cq*) of 40 cycles during the amplification reaction were considered not detectable. Crossing points between 5 and 40 cycles were considered detectable. The melting curves profiles were analyzed for every sample to detect unspecific products and primer dimers. Products were visualized on agarose gels, extracted and analyzed for sequence.

### 3.5. *In Vitro* Studies

For human PBMC isolation whole blood (25 mL) was drawn from healthy volunteers into sterile syringes containing 1.6 mg EDTA/mL blood. Whole blood was diluted 1:1 with PBS, layered on Ficoll (Biochrom, Berlin, Germany)-containing density gradient and centrifuged at 2500 RPM for 22 min. Afterwards the PBMC fraction was harvested and washed twice in PBS. 1 million cells per well were plated to 6 well-plates containing 2 mL RPMI with 1% FCS and 1% P/S. For each timepoint, three samples were stimulated with 500 ng/mL LPS (Invitrogen, Grand Island, NY, USA) or PBS. After 4, 12, 18 and 24 h cells were harvested and RNA was isolated with Pure Link RNA Mini Kit according to manufacture instructions. cDNA preparation was done with 1 μg per sample as described above.

For murine PBMC isolation six week old C57BL/6 mice were sacrificed and whole blood (1 mL per mouse) was drawn into 1.6 mg EDTA/mL containing syringes and pooled. The further steps were done as described for human PBMCs.

### 3.6. Histopathology

Kidney tissues were fixed in 4% neutral-buffered formalin, dehydrated in graded alcohols and embedded in paraffin. For periodic acid-Schiff (PAS) staining or immunostaining 4 μm sections were deparaffinized, rehydrated, transferred into citrate buffer, and either autoclaved or microwave treated for antigen retrieval and processed as described [70]. The following primary antibodies were used: anti-F4/80, anti-neutrophils (both Serotec, Oxford, UK) and anti-SMA (Dako, Glostrup, Denmark).

### 3.7. Statistics

Data were expressed as mean ± standard error of the mean (SEM). Comparison between groups was performed using univariate ANOVA (A value of *p* < 0.05 indicated statistical significance).

## 4. Conclusions

Together, we identified significant differences in the mRNA expression of PRR signaling regulators in human and mouse solid organs and in their regulation in transient inflammation and progressive tissue fibrosis. These findings can help to generate novel hypotheses on the role of single PRR signaling regulators in specific diseases. Furthermore, the species-specific expression of single PRR co-factors need to be considered in the interpretation of either data and human studies need to verify the functional roles of single factors suggested by studies performed in rodents.

## Supplementary Information



## Figures and Tables

**Table 1:** Human primers used for RT-PCR.

Human	Accession No.	Sequence
*A20*	NM_006290	forward primer: 5′-GGACTTTGCGAAAGGATCG-3′ reverse primer: 5′-TCACAGCTTTCCGCATATTG-3′
*CYLD*	NM_015247	forward primer: 5′-TTTGATGGAGTGCAGCTTTG-3′ reverse primer: 5′-CTCCTTTCCTGCGTCACACT-3′
*DUBA*	NM_017602	forward primer: 5′-GCAGGCTACAACAGTGAGGAC-3′ reverse primer: 5′-GCCTTTTCAAACCAATGCTC-3′
*ST2*	NM_003856	forward primer: 5′-CCCACTCAGGAAAGAAATCG-3′ reverse primer: 5′-TTCGCATATCCAGTCCTATTGA-3′
*CD180*	NM_005582	forward primer: 5′-CACCTCCTGGGATCAGATGT-3′ reverse primer: 5′-TGGTAGAGTGTCAGGGATTTCA-3′
*SIGIRR*	NM_021805	forward primer: 5′-CCCAGCTCTTGGATCAGTCT-3′ reverse primer: 5′-AGTCAGGGGCCCTATCACAG-3′
*TANK*	NM_133484	forward primer: 5′-CAAAGGAAGACTTGTAACCTGGA-3′ reverse primer: 5′-AGTTGCTCGCCAATGTTTTT-3′
*SOCS1*	NM_003745	forward primer: 5′-GACCCCTTCTCACCTCTTGA-3′ reverse primer: 5′-GTAGGAGGTGCGAGTTCAGG-3′
*SOCS3*	NM_003955	forward primer: 5′-GGAGACTTCGATTCGGGACC-3′ reverse primer: 5′-GAAACTTGCTGTGGGTGACC-3′
*SHIP*	NM_005541	forward primer: 5′-GTGACCCATCTGCAATACCC-3′ reverse primer: 5′-GGGTGGAGACACGACACTTT-3′
*IRAK-M*	NM_007199	forward primer: 5′-CTCGGTCATCTGTGGCAGTA-3′ reverse primer: 5′-TTCTAGGTGGGACCGGAAGT-3′
*DOK1*	NM_001381	forward primer: 5′-AGAGTCAGCGCTTTGGGAC-3′ reverse primer: 5′-CGACCCCTTATGGTCAAAGA-3′
*DOK2*	NM_003974	forward primer: 5′-GTACAGCAGCGCAGTCACAG-3′ reverse primer: 5′-AGCCCGGAGGGTATAGGAC-3′
*SHP1*	NM_080548	forward primer: 5′-CCCTCCCTACAGAGAGATGCT-3′ reverse primer: 5′-GAAGCTACCGTGGACACCTC-3′
*SHP2*	NM_002834	forward primer: 5′-GCGGGAGGAACATGACATC-3′ reverse primer: 5′-CGGAAAGTGTGAAGTCTCCAG-3′
*TOLLIP*	NM_019009	forward primer: 5′-GACAACTGTCTCCGTCGCA-3′ reverse primer: 5′-CGGGAGCTCACCGATGTA-3′
*IRF4*	NM_002460	forward primer: 5′-CCTGCAAGCTCTTTGACACA-3′ reverse primer: 5′-GAGTCACCTGGAATCTTGGC-3′
*SIKE*	NM_025073	forward primer: 5′-GTGGATGCTGAACCAGTCCT-3′ reverse primer: 5′-CCACCTGAACTGCTTTCCTC-3′
*NLRX1*	NM_024618	forward primer: 5′-CTGCCTCTGCTCTTCAACCT-3′ reverse primer: 5′-CTCGAAACATCTCCAGCACC-3′
*ERBIN*	NM_018695	forward primer: 5′-AATCATGTCAAGCGAAGCCT-3′ reverse primer: 5′-TGGGTTGAATTTATCTCCCTG-3′
*CENTB1*	NM_014716	forward primer: 5′-GCCTCTATTGAGCTGGTGGA-3′ reverse primer: 5′-ACTTTCCAGGAGACCAGTGC-3′
*Clec4a2*	NM_011999	forward primer: 5′-AGAGCTGGTTCATACAACATTGG-3′ reverse primer: 5′-TGACTTCCAATTCTTTGGGC-3′
*GAPDH*	NM_002046	forward primer: 5′-GAAGGTGAAGGTCGGAGTC-3′ reverse primer: 5′-GAAGATGGTGATGGGATTTC-3′
*18S*	NR_003278	forward primer: 5′-GCAATTATTCCCCATGAACG-3′ reverse primer: 5′-AGGGCCTCACTAAACCATCC-3′

**Table 2:** Murine primers used for RT-PCR.

Murine	Accession No.	Sequence
*A20*	NM_009397	forward primer: 5′-AAGCTCGTGGCTCTGAAAAC-3′ reverse primer: 5′-TTCCTCAGGACCAGGTCAGT-3′
*CYLD*	NM_173369	forward primer: 5′-GGGATGGAAGGTTTGATGG-3′ reverse primer: 5′-CTCCTTTCCTGTGTCACGCT-3′
*DUBA*	NM_138604	forward primer: 5′-AGCGGGCTACAACAGTGAAG-3′ reverse primer: 5′-AAGGCCTTTTCAAACCAGTG-3′
*ST2*	NM_010743	forward primer: 5′-TGACGGCCACCAGATCATTCACAG-3′ reverse primer: 5′-GCCAAAGCAAGCTGAACAGGCAATAC-3′
*CD180*	NM_008533	forward primer: 5′-GAGCCACCACATCCTCAGAT-3′ reverse primer: 5′-TGAGTTTGGTAAAGTGCCAGG-3′
*SIGIRR*	NM_023059	forward primer: 5′-GGATGACAAAGATCCCATGC-3′ reverse primer: 5′-ATGCAGATCCTGGTTTCCTG-3′
*TANK*	NM_011529	forward primer: 5′-GCTTCCAGAATGGGTACGTG-3′ reverse primer: 5′-TGGTAGGAATGCCAGCTCTC-3′
*SOCS1*	NM_009896	forward primer: 5′-ACTTCTGGCTGGAGACCTCA-3′ reverse primer: 5′-ACAAGCTGCTACAACCAGGG-3′
*SOCS3*	NM_007707	forward primer: 5′-AAGGCCGGAGATTTCGCT-3′ reverse primer: 5′-AACTTGCTGTGGGTGACCAT-3′
*SHIP*	NM_010566	forward primer: 5′-GCTGTTCCGGAATTGTGTTT-3′ reverse primer: 5′-GTGAAGAACCTCATGGGGAC-3′
*IRAK-M*	NM_028679	forward primer: 5′-CACTGCTGGGAGAGCTTTG-3′ reverse primer: 5′-CCAGCCAGCTGTTTGAAAGT-3′
*DOK1*	NM_010070	forward primer: 5′-TTTTCTGCCTTGGAGATGCT-3′ reverse primer: 5′-GCTCCAGGATTTGACTCTGC-3′
*DOK2*	NM_010071	forward primer: 5′-ATGGTCAGGATGGAGGAGC-3′ reverse primer: 5′-ATATAACACGGCTGCGAACC-3′
*SHP1*	NM_013545	forward primer: 5′-GTACCCACTGAACTGCTCGG-3′ reverse primer: 5′-ATCACCAGGTTGGCTGAGAC-3′
*SHP2*	NM_011202	forward primer: 5′-GACGGGAGGAACATGACATC-3′ reverse primer: 5′-AAAACTGCCATCGACTCCTC-3′
*TOLLIP*	NM_023764	forward primer: 5′-GCGGGTCTCTGTGCAGTT-3′ reverse primer: 5′-TGTGGGTGTTATACGGAGGAA-3′
*IRF4*	NM_013674	forward primer: 5′-TGCAAGCTCTTTGACACACA-3′ reverse primer: 5′-CAAAGCACAGAGTCACCTGG-3′
*SIKE*	NM_025679	forward primer: 5′-TTCAGGTGGACGATAACCAA-3′ reverse primer: 5′-GAGATTCACTGCTGATGGACAG-3′
*NLRX1*	NM_178420	forward primer: 5′-CACCTGGGTACCTTCGTGTT-3′ reverse primer: 5′-GCCCACAAATTCAACCACTT-3′
*ERBIN*	NM_021563	forward primer: 5′-GCCCTGAGACACCCTGAGA-3′ reverse primer: 5′-CAACCGCACAAACAAACTTC-3′
*CENTB1*	NM_153788	forward primer: 5′-CCTCGATTGAACTGGTGGAA-3′ reverse primer: 5′-AGGTAATGCTGTCCGCTCTC-3′
*Clec4a2*	NM_011999	forward primer: 5′-GCACAATGAATTGAACTGCAC-3′ reverse primer: 5′-GGAACCAAGTAGCAGTGGGA-3′
*GAPDH*	NM_008084	forward primer: 5′-CGTCCCGTAGACAAAATGGT-3′ reverse primer: 5′-TTGATGGCAACAATCTCCAC-3′
*18S*	NR_003278	forward primer: 5′-GCAATTATTCCCCATGAACG-3′ reverse primer: 5′-AGGGCCTCACTAAACCATCC-3′
