# Supplementary material for: Pattern-Recognition Receptor Signaling Regulator mRNA Expression in Humans and Mice, and in Transient Inflammation or Progressive Fibrosis
Source: Int J Mol Sci. 2013 Sep 4;14(9):18124–47. doi: 10.3390/ijms140918124 (PMC3794773; doi:10.3390/ijms140918124)
Supplement: Supplementary file 1 [file ijms-14-18124-s001.pdf]

Supplementary Information

Figure S1. Human PBMCs were stimulated with 10 ng/mL LPS. Detection of mRNA Expression levels after 4, 12, 18 and 24 h. Red colours illustrate increased expression, green colours illustrate decreased expression compared to controls.

LPS 10 ng/ml	A20	CYLD	DUBA	ST2	CD180	SIGIRR	TANK	SOCS1	SOCS3	SHIP	IRAK-M	DOK1	DOK2	SHP1	SHP2	TOLLIP	IRF4	SIKE	NLRX1	ERBIN	CENTB1	Clec4a2
0 hours	1.00	1.00	1.00	1.00	1.00	1.00	1.00	1.00	1.00	1.00	1.00	1.00	1.00	1.00	1.00	1.00	1.00	1.00	1.00	1.00	1.00	1.00
4 hours	3.37*	1.26	0.51	1.01	0.27*	0.60	1.37	1.03	4.97*	0.48*	0.82	0.45	0.14*	0.45	0.46	0.62*	0.98	0.58	0.42	1.90*	0.44	0.33
12 hours	2.00*	0.86	0.68	1.99	0.40*	0.79	2.21*	1.62*	7.16*	0.61	2.28*	0.55	0.11*	0.78	0.64	0.85	0.86	0.85	0.57	1.10	0.65	0.64
18 hours	1.44	1.04	0.66	1.19	0.34*	0.62	1.39	1.38	6.43*	0.49	1.42	0.45	0.09*	0.51	0.55	0.88	0.76	0.68	0.52	1.06	0.52	1.16
24 hours	1.01	1.12	1.04	1.02	0.66	1.13	1.30	1.75*	6.06*	0.87	1.97*	0.89	0.38*	1.27	1.09	1.19	1.33	1.14	1.01	0.96	1.16	1.70*
scale	0.20	0.50	1.00	2.00	5.00	10.00	20.00															

Table S1. Number of individual tissue samples used for human cDNA preparations.

**Human Organs**	**No. of individuals**
Lung	1
Liver	1
Kidney	4
Small Intestine	32
Colon	5
Testis	45
Spleen	3
Thymus	9
Brain	8
Heart	3

Table S2. (1) Descriptive statistics for murine healthy tissue housekeeping genes based on their crossing point (*C*p) values.

	**18S**	**GAPDH**
N	10	10
GM	9.73	17.65
AM	9.75	17.66
Minimum	8.81	16.54
Maximum	10.98	18.91
SD	0.74	0.77
Variance	0.55	0.59
CV	0.05	0.10

Table S2. (2) Descriptive statistics for human healthy tissue GAPDH based on their *C*p values.

	GAPDF
N	10
GM	25.76
AM	25.83
Minimum	22.69
Maximum	29.10
SD	2.02
Variance	4.08
CV	1.05

Table S2. (3) Descriptive stastistics of 18s housekeeping gene in IRI model based on *C*p values**.**

	**18S**
N	42
GM	10.11
AM	10.12
Minimum	8.99
Maximum	11.20
SD	0.48
Variance	0.23
CV	0.02

Table S2. (4) Descriptive stastistics of 18s housekeeping gene in LPS stimulations based on *C*p values in both species.

	**18S human**	**18S murine**
N	24	24
GM	12.28	11.06
AM	12.30	11.11
Minimum	10.94	9.38
Maximum	13.26	13.75
SD	0.61	1.09
Variance	0.38	1.18
CV	0.05	0.13
